# Alleviating Redox Imbalance Enhances 7-Dehydrocholesterol Production in Engineered *Saccharomyces cerevisiae*

**DOI:** 10.1371/journal.pone.0130840

**Published:** 2015-06-22

**Authors:** Wan Su, Wen-Hai Xiao, Ying Wang, Duo Liu, Xiao Zhou, Ying-Jin Yuan

**Affiliations:** 1 Key Laboratory of Systems Bioengineering (Ministry of Education), Tianjin University, Tianjin, China; 2 SynBio Research Platform, Collaborative Innovation Center of Chemical Science and Engineering (Tianjin), School of Chemical Engineering and Technology, Tianjin University, Tianjin, China; University of Huddersfield, UNITED KINGDOM

## Abstract

Maintaining redox balance is critical for the production of heterologous secondary metabolites, whereas on various occasions the native cofactor balance does not match the needs in engineered microorganisms. In this study, 7-dehydrocholesterol (7-DHC, a crucial precursor of vitamin D_3_) biosynthesis pathway was constructed in *Saccharomyces cerevisiae* BY4742 with endogenous ergosterol synthesis pathway blocked by knocking out the *erg5* gene (encoding C-22 desaturase). The deletion of *erg5* led to redox imbalance with higher ratio of cytosolic free NADH/NAD^+^ and more glycerol and ethanol accumulation. To alleviate the redox imbalance, a water-forming NADH oxidase (NOX) and an alternative oxidase (AOX1) were employed in our system based on cofactor regeneration strategy. Consequently, the production of 7-dehydrocholesterol was increased by 74.4% in shake flask culture. In the meanwhile, the ratio of free NADH/NAD^+^ and the concentration of glycerol and ethanol were reduced by 78.0%, 50.7% and 7.9% respectively. In a 5-L bioreactor, the optimal production of 7-DHC reached 44.49(±9.63) mg/L. This study provides a reference to increase the production of some desired compounds that are restricted by redox imbalance.

## Introduction

Maintaining redox balance plays an important role in the production of heterologous secondary metabolites [1] and cofactors act as redox carriers and energy transfer agents for the biochemical reactions [2]. However, the native cofactor balance does not match the needs in engineered microorganisms on various occasions [3]. Concretely, the intracellular redox potential is primarily determined by the ratio of NADH/NAD^+^ rather than that of NADPH/NADP^+^ [4]. When glycolytic NADH is produced beyond the cellular capacity for its oxidation, reduced products like ethanol and glycerol will be generated [4]. Imbalanced oxidation-reduction potential leads to cell damage, carbon or energy waste, and even metabolic arrest [5]. Cofactor engineering through different biotechnological techniques could reshape the whole-cell response to redox imbalance and optimize dynamic control of the target metabolic flux [5]. Six approaches are commonly involved in cofactor engineering, *i*.*e*. promoter engineering, gene-scale engineering, protein engineering, system metabolic engineering, structural synthetic biotechnology and cofactor regeneration [5,6].

Among the cofactor engineering approaches mentioned above, cofactor regeneration provides extra routes to alter the intracellular cofactor pool. After donating or accepting a functional group to or from other substrate of the enzymatic reaction, cofactors should be regenerated *in situ* by a separate reaction [7]. To be practical, chemical, electrochemical, photochemical and enzymatic methods are usually referred to for cofactor regeneration. However, the enzymatic methods are the most specific and compatible with the other components of enzymatic reactions [7]. Cofactor availability and the proportion of cofactor in the active form may dictate the overall process yield [8]. Two NADH oxidases, H_2_O-forming NADH oxidase (NOX) and alternative oxidase (AOX1), have been proved to be effective to alleviate the redox imbalance. Guo reported that constitutive expression of NOX in *Lactococcus lactis* rerouted pyruvate flux from lactate to diacetyl with an almost linear flux variation via altered NADH/NAD^+^ ratios [9]. Vemuri proved that the introduction of AOX1 and NOX into *Saccharomyces cerevisiae* reduced ethanol and glycerol formation by increasing the respiratory and non-respiratory NADH oxidation respectively [4]. Here the efficiency of cofactor regeneration was validated using the heterologous biosynthesis of 7-DHC in *S*.*cerevisiae* as an example.

7-DHC can be transformed into vitamin D_3_ after exposing to ultraviolet B radiation [10]. Traditionally, 7-DHC is produced by chemical transformation [11] and biological enzyme catalyzing reaction [12–14]. Recent attempt to *de novo* synthesizing 7-DHC by engineered yeast [15] provided a choice to avoid the disadvantages of traditional methods such as byproduct formation, costly substrate and environment pollution [16]. In order to produce 7-DHC in *S*. *cerevisiae* BY4742, the endogenous ergosterol synthesis pathway was blocked by knocking out the *erg5* gene (encoding C-22 desaturase) in this study. The deletion of *erg5* led to higher ratio of cytosolic free NADH/NAD^+^ and more accumulation of glycerol and ethanol than that in BY4742 strains, indicating the redox imbalance status to some extent. The production of 7-DHC was enhanced dramatically by the introduction of NOX [17] and AOX1 [18] based on cofactors regeneration. The ratio of free NADH/NAD^+^ and the accumulation of glycerol and ethanol were decreased in the mean while. This study provides a good example to enhance the production of some desired targeted products that are limited by redox imbalance.

## Materials and Methods

### Strains and media

The *S*.*cerevisiae* strains were grown at 30°C in YPD medium consisting of 2% (w/v) peptone, 1% (w/v) yeast extract and 2% (w/v) glucose. Engineered yeast strains were selected on synthetic complete (SD) medium: 0.67% (w/v) yeast nitrogen base, 2% (w/v) glucose with the appropriate amino acid drop out mix supplemented. *Escherichia coli* DH5α was used to maintain and amplify plasmids, which was cultured at 37°C in Luria-Bertani (LB) broth. Ampicillin (100 μg/ml) was added to the media prior to use.

### DNA manipulation and plasmids construction

All oligonucleotides used in this study are listed in Table 1. Each promoter or terminator was amplified from the genomic DNA of *S*.*cerevisiae* BY4742 [19]. The endogenous genes *adh2*, *ald6* and *thmg1* (a truncated *hmg1* gene) were PCR amplified from *S*.*cerevisiae* BY4742 chromosomal DNA according to their sequence information published in Saccharomyces Genome Database (http://www.yeastgenome.org). The *Salmonella enterica* acetyl-CoA synthetase (ACS) with L641P mutation, the mus musculus ATP-citrate lyase (ACL) and the *Homo sapiens* C-24 reductase (DHCR24) were codon optimized (S1 Fig) and synthesized by Genewiz Inc. The water-forming NADH oxidase (NOX) from *Streptococcus pneumoniae* and the alternative oxidase (AOX1) from *Histoplasma capsulatum* were synthesized by Genscript Inc. according to their sequences published in genbank (Accession no. AF014458 and AF133236). The mannitol-1-phosphate 5-dehydrogenase gene *mtlD* from *E*.*coli* was obtained by colony PCR of DH5α. The bleomycin resistance gene *bleMX* together with its promoter and terminator was cloned from the plasmid pBLE owned by our laboratory.

**Table 1:** Primers used in this study.

Oligo	Sequence (5’ to 3’)
**FBA1p-pRS425-F**	cctcgaggtcgacggtatcgataagcttgatatcgaattctccaactggcaccgctggct
**FBA1p-tHMG1-R**	aagacttcttggtgacttcagttttcaccaattggtccattttgaatatgtattacttgg
**tHMG1-FBA1p-F**	tttgtcatatataaccataaccaagtaatacatattcaaaatggaccaattggtgaaaac
**tHMG1-HXT7t-R**	aattagagcgtgatcatgaattaataaaagtgttcgcaaattaggatttaatgcaggtga
**HXT7t-tHMG1-F**	tcgtttgaaagatgggtccgtcacctgcattaaatcctaatttgcgaacacttttattaa
**HXT7t-ENO2p-R**	atagagtaaagaaccctttctatacccgcagcgtcgacacataactgactcattagacac
**ENO2p-HXT7t-F**	tctgtatcccgcttcaaaaagtgtctaatgagtcagttatgtgtcgacgctgcgggtata
**ENO2p-DHCR24-R**	acaacaaagcgcagactgccaaagagacagcaggttccattattattgtatgttatagta
**DHCR24-ENO2p-F**	tcataacaccaagcaactaatactataacatacaataataatggaacctgctgtctcttt
**DHCR24-ADH1t-R**	tggagacttgaccaaacctctggcgaagaagtccaaagcttcaatgtctagctgccttac
**ADH1t-DHCR24-F**	tgaggtttacgacaagatttgtaaggcagctagacattgaagctttggacttcttcgcca
**ADH1t-pRS425-R**	gctggagctccaccgcggtggcggccgctctagaactagtcatgccggtagaggtgtggt
**PGI1p-pRS413-F**	gccccccctcgaggtcgacggtatcgataagcttgatatcggtgggtgtgggtgtattgg
**PGI1p-ADH2-R**	cgtagaagataatggctttttgagtttctggaatagacatttttaggctggtatcttgat
**ADH2-PGI1p-F**	tcttgcaaaatcgatttagaatcaagataccagcctaaaaatgtctattccagaaactca
**ADH2-GPM1t-R**	ggagggaaaaagaaatcatcaaatcattcattcttcagacttatttagaagtgtcaacaa
**GPM1t-ADH2-F**	ccaaattgctggtagatacgttgttgacacttctaaataagtctgaagaatgaatgattt
**GPM1t-PGK1p-R**	atatctgtgcgtcttgagttgaagtcaggaatctaaaatatattcgaactgcccattcag
**PGK1p-GPM1t-F**	accaattgcaaagggaaaagctgaatgggcagttcgaatatattttagattcctgacttc
**PGK1p-ALD6-R**	tcttgactggttcagcagtgtcaaagtgtagcttagtcattgttttatatttgttgtaaa
**ALD6-PGK1p-F**	aaggaagtaattatctactttttacaacaaatataaaacaatgactaagctacactttga
**ALD6-GPDt-R**	aagaaaatttatttaaatgcaagatttaaagtaaattcacttacaacttaattctgacag
**GPDt-ALD6-F**	tgcatacactgaagtaaaagctgtcagaattaagttgtaagtgaatttactttaaatctt
**GPDt-PDC1p-R**	cacatcacatcagcggaacatatgctcacccagtcgcatgggaatctgtgtatattactg
**PDC1p-GPDt-F**	agataacatatatctagatgcagtaatatacacagattcccatgcgactgggtgagcata
**PDC1p-ACS-R**	cgatatttgcaggaatggcgtgcttgtgtgtctgagacattttgattgatttgactgtgt
**ACS-PDC1p-F**	tcataacctcacgcaaaataacacagtcaaatcaatcaaaatgtctcagacacacaagca
**ACS-FBA1t-R**	aatactcattaaaaaactatatcaattaatttgaattaacttaagaaggcattgctatgg
**FBA1t-ACS-F**	gccattggaggaaaagcaagccatagcaatgccttcttaagttaattcaaattaattgat
**FBA1t-TPI1p-R**	acgggtaatcttccaccaacctgatgggttcctagatataaaagatgagctaggcttttg
**TPI1p-FBA1t-F**	tacaacgtaagatatttttacaaaagcctagctcatcttttatatctaggaacccatcag
**TPI1p-ACL-R**	attccttaccagtttgttcagagatagccttagcagacatttttagtttatgtatgtgtt
**ACL-TPI1p-F**	ttaaatctataactacaaaaaacacatacataaactaaaaatgtctgctaaggctatctc
**ACL-PGK1t-R**	aaagaaaaaaattgatctatcgatttcaattcaattcaatttacatagacatgtgttctg
**PGK1t-ACL-F**	cgacatctcttacgttttgccagaacacatgtctatgtaaattgaattgaattgaaatcg
**PGK1t-pRS413-R**	gctggagctccaccgcggtggcggccgctctagaactagtaacgaacgcagaattttcga
**TPI1p-pRS416-F**	gccccccctcgaggtcgacggtatcgataagcttgatatctatatctaggaacccatcag
**TPI1p-NOX-R**	taccagcgtggttagcaccgactacaacgattttactcatttttagtttatgtatgtgtt
**NOX-TPI1p-F**	ttaaatctataactacaaaaaacacatacataaactaaaaatgagtaaaatcgttgtagt
**NOX-TEF1t-R**	aagatatgcaactagaaaagtcttatcaatctccttatttttatttttcagccgtaaggg
**TEF1t-NOX-F**	caactacatcacaatggctgcccttacggctgaaaaataaaaataaggagattgataaga
**TEF1t-GPM1p-R**	ttcctgctcacaaatcttaaagtcatacattgcacgactagatagcgccgatcaaagtat
**GPM1p-TEF1t-F**	attcgatattgtcgtaacaaatactttgatcggcgctatctagtcgtgcaatgtatgact
**GPM1p-AOX1-R**	gctgtgatggaagtgaggtgtattagtaatggcagtgctgtattgtaatatgtgtgtttg
**AOX1-GPM1p-F**	attcttcttaataatccaaacaaacacacatattacaatacagcactgccattactaata
**AOX1-TPI1t-R**	aaagaaaagaagataatatttttatataattatattaatcgttttgtttaagctgatgca
**TPI1t-AOX1-F**	cttcacggccggcaaaaaattgcatcagcttaaacaaaacgattaatataattatataaa
**TPI1t-pRS416-R**	gctggagctccaccgcggtggcggccgctctagaactagttatataacagttgaaatttg
**TPI1p-F(NotI)**	*gcggcc*gctatatctaggaacccatcaggttgg
**TPI1p-mtlD-R**	cacgaccgatattacctgcgccaaaatgtaatgctttcatttttagtttatgtatgtgtt
**mtlD-TPI1p-F**	ttaaatctataactacaaaaaacacatacataaactaaaaatgaaagcattacattttgg
**mtlD-PGK1t-R**	aaagaaaaaaattgatctatcgatttcaattcaattcaatttattgcattgctttataag
**PGK1t-mtlD-F**	tgtatccgaggcggtaaccgcttataaagcaatgcaataaattgaattgaattgaaatcg
**PGK1t-R(NotI)**	*gcggcc*gcaacgaacgcagaattttcgagttat
**bleMX-F**	agatctgtttagcttgcctcgtccccgccg
**bleMX-TPI1p-R**	acgggtaatcttccaccaacctgatgggttcctagatataattaagggttctcgagagct
**TPI1p-bleMX-F**	gccatccagtgtcgaaaacgagctctcgagaacccttaattatatctaggaacccatcag
**PGK1t-ERG5DN-R**	aatatgatttattgtctggacaaagttctgtttttccccaaacgaacgcagaattttcga
**ERG5DN-PGK1t-F**	cgtattttaagtttaataactcgaaaattctgcgttcgtttggggaaaaacagaactttg
**ERG5DN-R**	gcatccactggagaagaacatccttcttgg

Homology arms were underlined and the restriction site *Not*I was italic.

**Fig 1:**
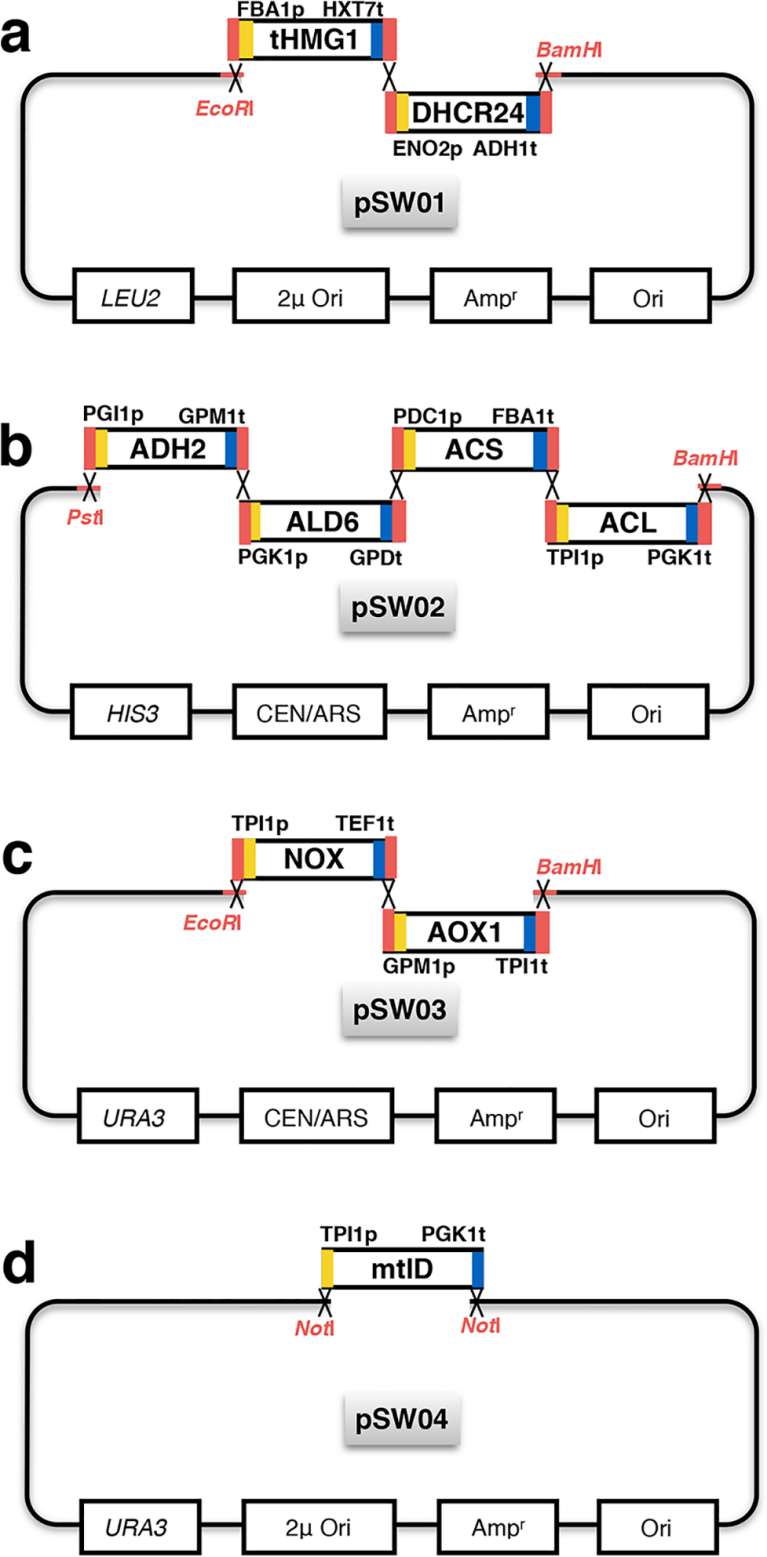
Plasmids construction *via* RADOM method. The red bars represent the homologous regions between different DNA fragments and vectors. The yellow and blue bars represent the promoter and terminator of each gene. The restriction sites are emphasized in red. (a) Plasmid pSW01 contains truncated HMG-CoA reductase (tHMG1) encoding gene and C-24 reductase (DHCR24) encoding gene. (b) Plasmid pSW02 contains alcohol dehydrogenase (ADH2) encoding gene, acetaldehyde dehydrogenase (ALD6) encoding gene, acetyl-CoA synthetase (ACS) variant encoding gene and ATP-dependent citrate lyase (ACL) encoding gene. (c) Plasmid pSW03 contains water-forming NADH oxidase (NOX) encoding gene and alternative oxidase (AOX1) encoding gene. (d) Plasmid pSW04 contains mannitol-1-phosphate 5-dehydrogenase encoding gene *mtlD*.

Each individual gene expression cassette was assembled by overlap extension PCR (OE-PCR) and gel purified by TIANgel Midi Purification Kit (TIANGEN Biotech, DP209). The resulting fragment TPI1p-*mtlD*-PGK1t was digested with *Not*I, and inserted into the corresponding site of pRS426. Other gene expression cassettes sharing 40bp homologous regions with the adjacent fragments or linearized vector were assembled based on RADOM method [20] (Fig 1). The correct clones were verified by PCR verification and restriction enzyme digestion. To integrate the *mtlD* into the genome of the strains, three fragments pAgTEF1-*bleMX*-tAgTEF1, TPI1p-*mtlD*-PGK1t and ERG5-down were obtained by OE-PCR. All plasmids and engineered yeast strains constructed were listed in Table 2 and Table 3.

**Table 2:** Plasmids used in this study.

Plasmid	Description
**pRS425**	Multiple copy plasmid in *S*.*cerevisiae* with *LEU*2 marker* *
**pRS426**	Multiple copy plasmid in *S*.*cerevisiae* with *URA*3 marker* *
**pRS413**	Single copy plasmid in *S*.*cerevisiae* with *HIS*3 marker
**pRS416**	Single copy plasmid in *S*.*cerevisiae* with *URA*3 marker
**pSW01**	pRS425 with *thmg1* and *dhcr24* (*Homo sapiens*, codon optimized)
**pSW02**	pRS413 with *adh2*, *ald6*, *acs* ^*L641P*^ (*Salmonella enterica*, codon optimized) and *acl* (mus musculus, codon optimized)
**pSW03**	pRS416 with nox (*Streptococcus pneumoniae)* and *aox1* (*Histoplasma capsulatum*)
**pSW04**	pRS426 with *mtlD* (*Escherichia coli*)
**pbleMX**	pRS425 with *bleMX*

**Table 3:** Yeast strains used in this study.

Strain	Description
**BY4742**	MATα *his3Δ1 leu2Δ0 lys2Δ0 ura3Δ0 *
**ΔERG5**	MATα *his3Δ1 leu2Δ0 lys2Δ0 ura3Δ0 erg5Δ*:: *kanMX*
**SyBE_Sc01100001**	BY4742 with plasmid pSW01
**SyBE_Sc01100002**	ΔERG5 with plasmid pSW01
**SyBE_Sc01100010**	BY4742 with plasmids pSW01 and pSW02
**SyBE_Sc01100011**	ΔERG5 with plasmids pSW01 and pSW02
**SyBE_Sc01100020**	ΔERG5 with plasmids pSW01, pSW02 and pSW03
**SyBE_Sc01100021**	BY4742 with plasmid pSW04
**SyBE_Sc01100022**	ΔERG5 with plasmid pSW04
**SyBE_Sc01100023**	BY4742 with plasmids pSW01 and pSW04
**SyBE_Sc01100024**	ΔERG5 with plasmids pSW01 and pSW04
**SyBE_Sc01100025**	BY4742 with plasmids pSW01, pSW02 and pSW04
**SyBE_Sc01100026**	ΔERG5 with plasmids pSW01, pSW02 and pSW04
**SyBE_Sc01100027**	MATα *his3Δ1 leu2Δ0 lys2Δ0 ura3Δ0 erg5Δ*::*bleMX*-TPI1p-*mtlD*-PGK1t
**SyBE_Sc01100028**	SyBE_Sc01100027 with pSW01 and pSW02
**SyBE_Sc01100029**	SyBE_Sc01100027 with pSW01, pSW02 and pSW03

### Shake flask culture and bioreactor fermentation procedures

For shake flask culture, a single colony of engineered yeast from plate was cultivated in a glass tube containing 4 ml appropriate SD medium at 30°C, 220 rpm for 24 h. And then all the pre-culture was inoculated into a 250 ml conical flask containing 50 mL corresponding SD.After 12 h cultivation, the seed almost entered mid-exponential phase and was transferred to 100 ml fresh YPD medium at an initial OD_600_ of 0.2. The cultures were grown in the same conditions mentioned above until harvest.

For large-scale culture in 5-L stirred-tank bioreactor, 10% (v/v) seed culture was transferred to 2.5 L of YPD medium. Concentrated glucose solution (50%, w/v) was fed periodically to keep the concentration of the glucose under 2.0 g/L. The temperature, pH, dissolved oxygen and airflow were controlled at 30°C, 5.5, >30% and 1.5 vvm, respectively. Duplicate samples were collected to determine the cell density, glucose concentration and the 7-DHC production. To avoid the spontaneous degradation of 7-DHC, all the shaking incubator and bioreactor were located at a dark room.

### Analysis of cell growth, glycerol, ethanol and acetyl-CoA

During the fermentation, the OD at 600 nm was measured with a spectrophotometer to monitor the cell growth. For HPLC analysis, all samples were centrifuged at 12000 rpm for 1 min. The supernatants were filtered through a 0.22 μm filter and diluted prior to use. Glucose, ethanol and glycerol were separated on an Aminex HPX-87H ion-exchange column (Bio-Rad, Hercules, CA, USA) and detected by Waters 2414 refractive index detector. The temperatures of the column oven and detector were kept at 65°C and 50°C respectively. 5 mM sulfuric acid was chosen as the mobile phase with a flow rate of 0.5 ml/min.

Acetyl-CoA measurements were conducted with the PicoProbe Acetyl-CoA Quantification Kit (BioVision, San Francisco, CA, USA). As described in the instruction, samples were frozen rapidly in liquid nitrogen and pulverized, and then the precooled 1N perchloric acid was added into the fine cell powder and homogenized by vortex. The following procedures were the same as the protocols provided by the manufacturer. Furthermore, all procedures were conducted on ice to avoid the degradation of acetyl-CoA.

### Measurement of cytosolic free NADH/NAD^+^ ratio

Canelas *et al*. (2008) [21] reported that local free NAD^+^/NADH ratio could be determined from product/substrate ratios of suitable near-equilibrium redox reactions. When the bacteria mannitol-1-phosphate 5-dehydrogenase was expressed in *S*.*cereviaise*, the cytosolic free NAD^+^/NADH ratio was measured from [fructose-6-phosphate]/[mannitol-1-phosphate] ratio. The mannitol-1-phosphate 5-dehydrogenase gene (*mtlD*) from *E*.*coli* was introduced into engineered strains from BY4742 and ΔERG5 for free NAD/NADH ratio measurement. Leakage-free quenching was performed according to Canelas *et al* [22]. The metabolite extraction process was based on Wang *et al*. [23] with minor modifications. After collecting the quenched cells by centrifugation, the pellet was washed with ice-cold Milli-Q double deionized water for three times and then ground into fine powder in liquid nitrogen. 1 ml 50% methanol/water (v/v, -40°C) was used to suspend 50 mg cell powders and frozen in liquid nitrogen and thawed for three times. After centrifugation to collect the supernatant, another 500 μl 50% methanol/water was added to the cells to extract the metabolites further and combined with the former 1 ml extraction liquor. Fructose-6-phosphate and mannitol-1-phosphate were analyzed by LC-MS according to Kato *et al*. [24].

### 7-dehydrocholesterol production analysis

The yeast cells were harvested by centrifugation at 5000 rpm for 2 min. Then, the pellets were washed once with Milli-Q double deionized water. Cells were ground into fine powder in liquid nitrogen and then suspended in 5 ml 1.5 M KOH-methanol solution. After keeping at 80°C for 90 min, 2 ml n-hexane were added into the tube for 7-DHC extraction with thorough vortex. The organic phase was collected by centrifugation at 12000 rpm for 2 min and then was freeze dried for 30 min. 400 μl n-hexane was added to re-suspend the product and refreeze dried overnight. The derivatization of the freeze-dried product was conducted with 200 μl N-methyl-N-(trimethylsilyl) trifluoroacetamide (MSTFA) at 30°C for 2 h. All centrifugal tubes used for 7-DHC extraction were covered with aluminum foil to avoid exposure to UV-light. To verify the extraction process, different amounts of 7-DHC standards were added into the wild-type BY4742 yeast cells before ground in liquid nitrogen and the average recovery ratio is 82.4%±3.7% in five batch independent extraction operations.

For GC-TOF-MS analysis, the samples were diluted with n-hexane and injected into an Agilent 6890 gas chromatograph coupled to Waters time-of-flight mass spectrometry. The gas chromatograph was equipped with a DB-5 fused-silica capillary column (30 m * 0.25 mm i.d., film thickness 0.25 μm, J&W Scientific, Folsom, CA). Helium was used as carrier gas. Ions were generated by a 70 eV electron beam in EI mode at an ionization current of 40 μA. Mass spectra were acquired at a range of 50–800 m/z. The ion source temperature was 250°C and the injection site temperature was 260°C. The column temperature was initially maintained at 70°C for 2 min, then increased to 250°C at a temperature ramp of 30°C/min, followed by an increase to 280°C at a temperature ramp of 10°C/min and maintained at 280°C for 15min, finally increased to 290°C at a temperature ramp of 5°C/min and maintained at 290°C for 5 min.

### The statistical analysis

All statistical analyses were performed with software SPSS 19.0 and the level of significance was set at *P*<0.05. Data were collected from at least three replicate samples.

## Results

### Blocking ergosterol biosynthesis pathway was essential for 7-DHC production

**Fig 2:**
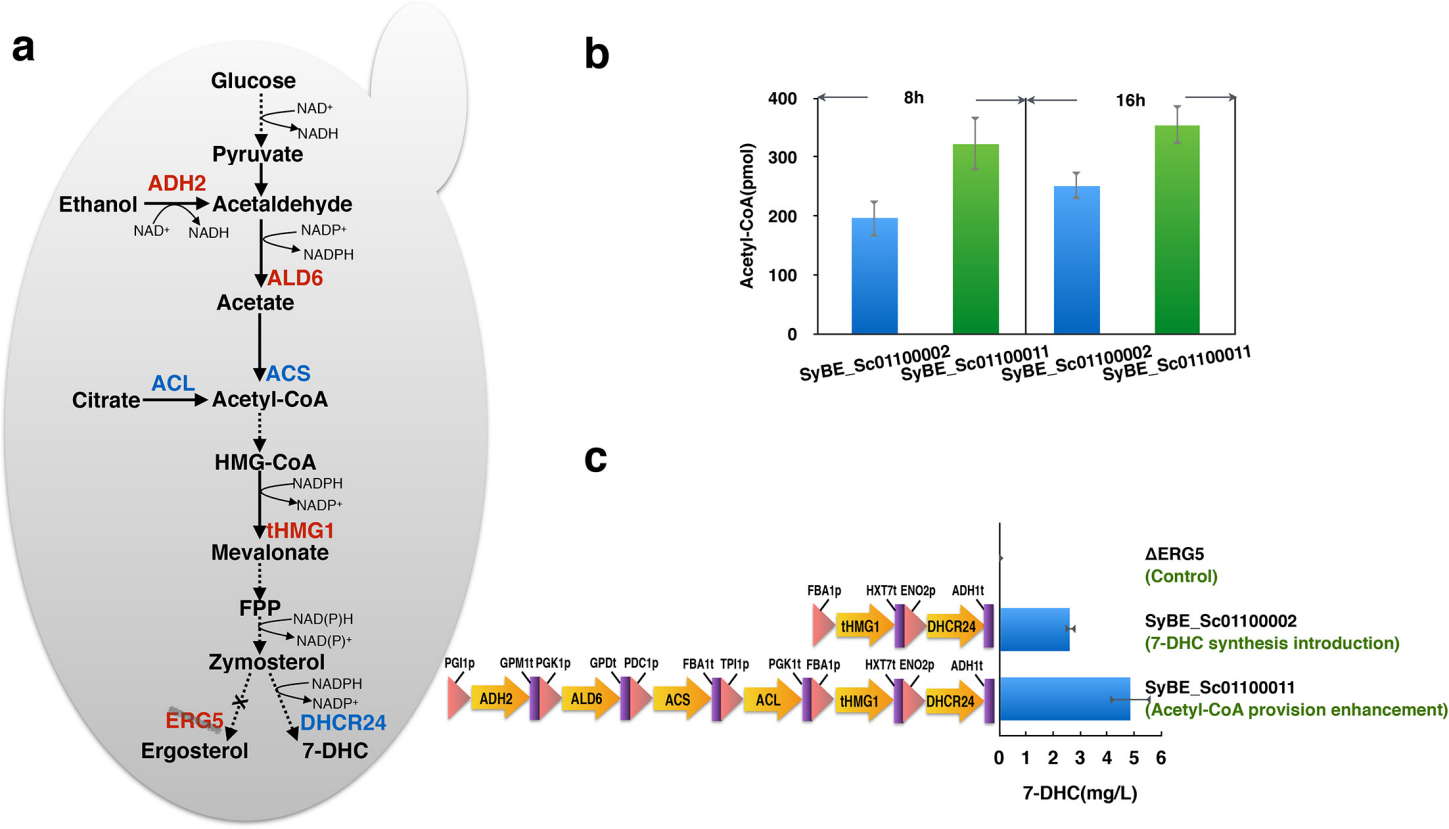
Genetic modification in 7-DHC producing engineered yeast strains. (a) Genetic modification of the wild type BY4742 strain including the disruption of ergosterol synthesis (*erg5* deletion), the construction of the 7-DHC biosynthesis (the introduction of *dhcr24* and the over-expression of *thmg1*) and the enhancement of acetyl-CoA provision (the over-expression of *adh2* and *ald6* together with the introduction of *acs* and *acl*). Genes in red represent endogenous genes and genes in blue represent heterologous genes. (b) Intracellular acetyl-CoA in SyBE_Sc01100002 (before the enhancement in acetyl-CoA provision) and SyBE_Sc01100011 (after the enhancement in acetyl-CoA provision) was quantified in the mid-logarithmic phase (8 h) and stationary phase (16 h). (c) The amount of 7-dehydrocholesterol produced by engineered yeast was measured after 36 h shake flask culture.

The *dhcr24* gene encodes a FAD-dependent oxidoreductase, which catalyzes the reduction of the delta-24 double bond of sterol intermediates [15]. The enzyme 3-hydroxy-3-methylglutaryl-coenzyme-A (HMG-CoA) reductase is known as the rate- limiting enzyme in early sterol biosynthesis of eukaryotic cells. A truncated form of the *hmg1* gene, which encodes a protein that contains the catalytically active domain in C-terminal but lacks the N-terminal membrane-spanning region is proved to be effective to overcome the regulatory effects and to avoid karmella formation [25]. To construct a functional 7-DHC biosynthesis pathway, a *Homo sapiens* C-24 reductase (DHCR24) was introduced and the endogenous HMG-CoA reductase without membrane-binding region (tHMG1) was over-expressed in *S*.*cerevisiae* BY4742, obtaining SyBE_Sc01100001 (Fig 2A). However, ergosterol rather than 7-DHC was observed as the main product, indicating the natural ergosterol biosynthesis pathway competed much with 7-DHC formation pathway [26]. Thus the *S*.*cerevisiae* BY4742 *erg5* null mutant strain ΔERG5 was then chosen as chassis in this study to eliminate the competition of ergosterol biosynthesis pathway, and the result of GC-TOF-MS showed no detection of ergosterol (S2 Fig) accordingly. When the *dhcr24* gene and *thmg1* gene were introduced into ΔERG5 strain, obtaining SyBE_Sc01100002, a titer of 2.61±0.19 mg/L 7-DHC was obtained in this strain (Fig 2A and 2C).

### Additional supply of acetyl-CoA in cytosol led to an increase in 7-DHC production

Acetyl-CoA is the central metabolite in carbon and energy metabolism [27], especially acts as the key precursor for mevalonate pathway in *S*.*cerevisiae*, which provides backbone for a wide range of secondary metabolites [28]. The ethanol degradation pathway, together with the pyruvate dehydrogenase bypass improves provision of acetyl-CoA in cytoplasm [29,30]. *adh2* and *ald6* encode alcohol dehydrogenase and acetaldehyde dehydrogenase in yeast cells. The overexpression of the endogenous ADH2 and ALD6, combined with a codon-optimized ACS vatiant (L641P) from *S*. *enterica*, channels the carbon flux from ethanol to acetyl-CoA [31] and increases the yield of isoprenoids and fatty acid ethyl esters in *S*.*cerevisiae* [31,32]. In addition, *acl* encodes ATP-dependent citrate lyase, catalyzing the reaction from citrate to acetyl-CoA in oleaginous yeasts [33]. Tang reported that ACL increased the accumulation of acetyl-CoA in cytosol for fatty acid synthesis [34]. Lian has proved that the combined strategies of the over-expression of ADH2, ALD6, ACS and ACL to direct the glycolytic flux towards acetyl-CoA resulted in an improvement in n-butanol production [16]. After integrating two pathways to direct the carbon fluxes to acetyl-CoA (Fig 2A), the engineered yeast SyBE_Sc01100011 showed enriched acetyl-CoA content compared with SyBE_Sc01100002. To be specific, the amounts of acetyl-CoA in SyBE_Sc01100011 were 64.29% (in mid-logarithmic phase) and 41.04% (in stationary phase) more than those in SyBE_Sc01100002. The highest amount of acetyl-CoA (354 pmol) was detected in SyBE_Sc01100011 after 16 h culture (Fig 2B). The extra supply of acetyl-CoA in cytosol led to an increase in 7-DHC production by 85.44% in SyBE_Sc01100011 compared with SyBE_Sc01100002 (Fig 2C).

### The deletion of *erg5* led to redox imbalance in 7-DHC production strains

**Fig 3:**
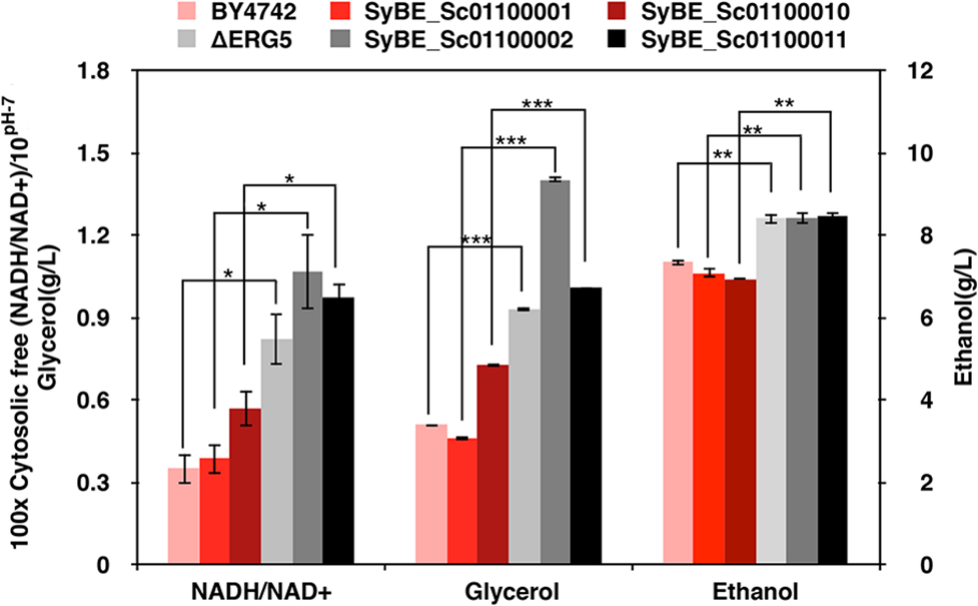
The cytosolic free NADH/NAD^+^ ratio and metabolites accumulation in strains from BY4742 and the ergosterol defected strains (ΔERG5) strains. All strains were cultivated in shake flasks and sampled after 24 h after they were inoculated into the fresh YPD medium. Columns with different shades of red color represent the strains from the wild type BY4742 and those with different shades of gray color represent the strains from ΔERG5. Significance levels of Students t-test: *P< 0.05, **P< 0.01, ***P< 0.001.

During the fermentation in shake flasks, the cytosolic free NADH/NAD^+^ ratio and the amounts of glycerol and ethanol produced by strains derived from BY4742 and ΔERG5 were measured at 24 h in the stationary phase. The free NADH/NAD^+^ ratios and glycerol and ethanol accumulation in the ergosterol defected strains were higher than those of the strains from BY4742 with the same alterations. Especially, the free NADH/NAD^+^ ratio of SyBE_Sc01100002 was 0.011(±0.003), while the ratio of strains engineered from BY4742 were less than half of that value. At the same time, the glycerol produced by SyBE_Sc01100002 reached 1.402 g/L, which was the highest among all yeast strains in this study (Fig 3). Because excessive NADH in cytosol gave rise to the redox imbalance, decreasing the ratio of NADH/NAD^+^ by cofactor engineering became a rational approach to alleviate redox imbalance in the ergosterol defected strains.

### Alleviating the redox imbalance *via* NADH regeneration system increased the production of 7-DHC

**Fig 4:**
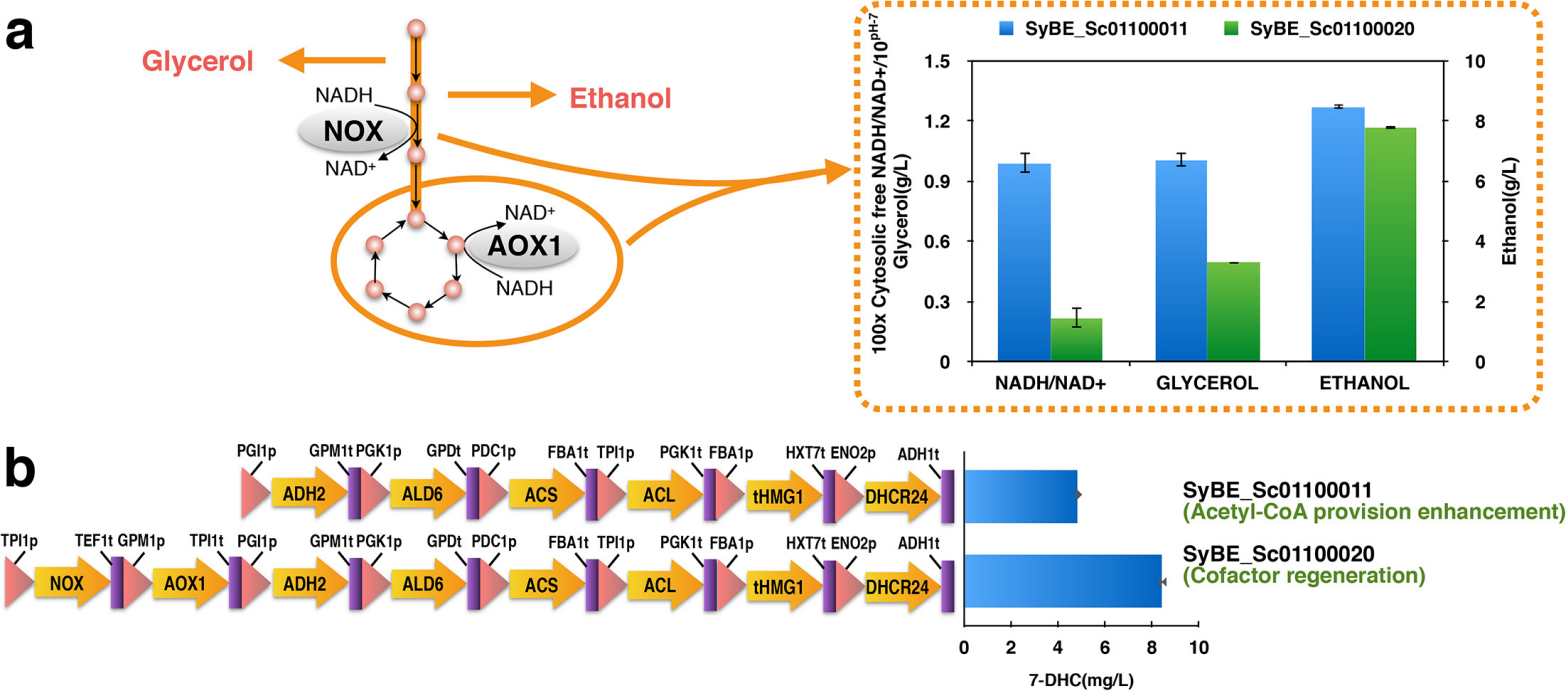
Effects of NADH regeneration system on 7-DHC production, by-product accumulation and cytosolic free NADH/NAD^+^ ratio. (a) Cofactor regeneration system consisted of the H_2_O-forming NADH oxidase (NOX) and the alternative oxidase (AOX1). The NOX localizes in the cytosol, whereas AOX1 is directed to the mitochondria. NOX primarily impacts glycerol production, while AOX1 influences ethanol formation. SyBE_Sc01100011 was ΔERG5 with the 7-DHC biosynthesis pathway (the introduction of *dhcr24* and the over-expression of *thmg1*) and the acetyl-CoA provision enhancement module (the over-expression of *adh2* and *ald6* together with the introduction of *acs* and *acl*). SyBE_Sc01100020 was SyBE_Sc01100011 with the cofactor regeneration system (the introduction of *nox* and *aox1*). All strains were cultivated in shake flasks and sampled after 24h after they were inoculated into the fresh YPD medium. (b) 7-DHC production was measured after 36 h shake flask culture.

**Fig 5:**
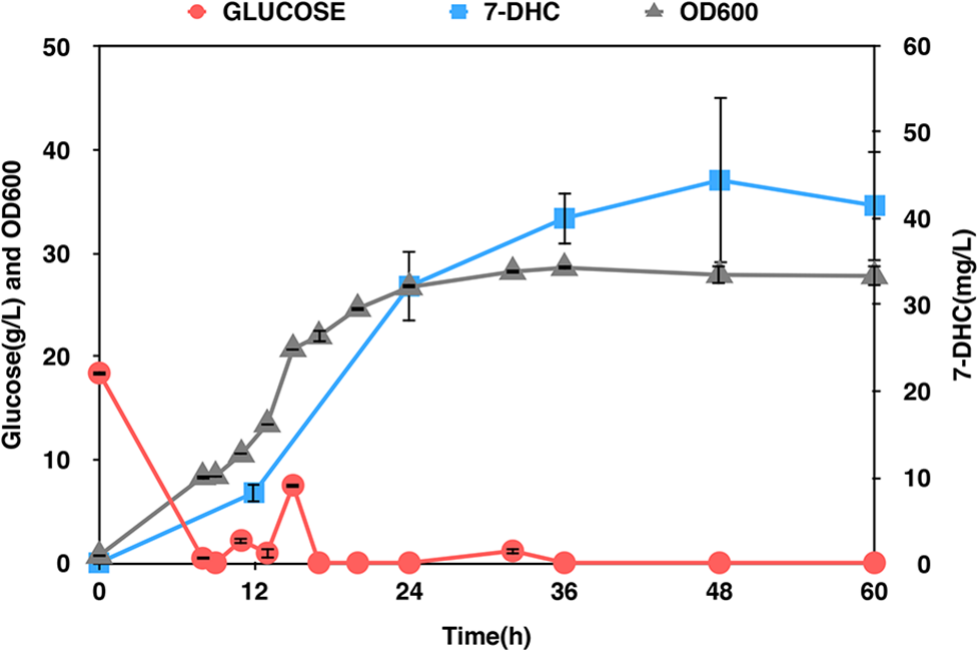
Optimization of the 7-DHC production in bioreactor. SyBE_Sc01100020 was cultivated in a 5-L bioreactor and the glucose was controlled under a concentration of 2 g/L. The red line stands for the concentration of glucose, the gray line stands for the cell density and the blue line stands for the production of 7-DHC.

In this study, the NADH regeneration system consisted of two NADH oxidases: NOX and AOX1 (Fig 4A). H_2_O-forming NADH oxidase (NOX) purified from *Streptococcus pneumoniae* is a soluble flavoprotein that reoxidizes NADH and reduces molecular O_2_ to water [17]. *Histoplasma capsulatum* alternative oxidase (AOX1) possesses cyanide-insensitive oxygen-consuming activities [18]. When the heterologous NADH oxidases were expressed in *S*. *cerevisiae*, the NOX localized in the cytosol, whereas AOX1 was directed to the mitochondria [4]. NOX primarily impacts glycerol production by mediating the non-respiratory dissipation of NADH [4]. The glycerol synthesis pathway is activated in *S*. *cerevisiae* as the outlet for NADH consumption [35] and NOX relieves the need to generating glycerol [4]. On the other hand, aerobic ethanol formation in *S*. *cerevisiae* is the result of a limitation in electron transport from NADH to oxygen [36]. AOX1 up-regulates almost every step of TCA cycle and recruits parts of the respiratory system in the transfer of electrons from NADH to oxygen [4]. Both of these two NADH oxidases were incorporated into SyBE_Sc01100011 to obtain SyBE_Sc01100020 in our study. Consequently, the cytosolic free NADH/NAD^+^ ratio and the concentration of glycerol and ethanol were reduced by 78.0% (from 0.010±0.001 to 0.002±0.001), 50.7% (from 1.006±0.003 g/L to 0.496±0.003 g/L) and 7.9% (from 8.463±0.077 g/L to 7.788±0.035 g/L) respectively (Fig 4A). The production of 7-DHC in SyBE_Sc01100020 was increased by 74.4% (from 4.84±0.75 mg/L to 8.44±0.42 mg/L) (Fig 4B). And when the SyBE_Sc01100020 strain was cultivated in a 5-L bioreactor under glucose restriction strategy (<2 g/L), the production of 7-DHC reached 44.49±9.63 mg/L, which was the highest reported titer as known so far (Fig 5).

## Discussion

As a major sterol in *S*.*cerevisiae*, ergosterol is required for the normal structure and function of cellular membranes *via* regulating the delicate balance among membrane components such as lipids and proteins [37,38]. In order to produce vertebrate sterol 7-DHC in *S*.*cerevisiae*, the natural ergosterol biosynthesis pathway should be blocked to avoid competition for limited carbon sources. Nevertheless, the ergosterol defected yeast strains (strains engineered from ΔERG5) showed a more reduced cell environment with higher NADH/NAD^+^ ratio compared with those derived from the wild-type BY4742 with the same modifications (Fig 3). Ergosterol defect may activate some transcription factors such as Upc2 [39] to enhance the expression of related genes in 7-DHC synthesis pathway, which might be the reason for redox imbalance in ΔERG5 strains. Based on the theoretical stoichiometry in 7-DHC biosynthesis pathway, it shows that it consumes 9 molecules glucose per 7-DHC and generates 21 molecules NADH (S3 Fig and Eq 1), indicating the introduction of 7-DHC biosynthesis pathway could bring redox imbalance. As shown in Fig 3, the cytosolic free NADH/NAD^+^ ratio in SyBE_Sc01100002 was indeed significant higher than that in ΔERG5.

$$9\;\text{Glucose}+18\;\text{ATP}+6\;{\text{O}}_{2}+\text{NADPH}\to 1\;7-\text{DHC}+26\;{\text{CO}}_{2}+1\;\text{Formate}+11\;{\text{H}}_{2}\text{O}+21\;\text{NADH}+18\;\text{AMP}+18\;\text{PPi}$$ (1)

7-DHC and ergosterol shares most of the biosynthesis pathway [40], any other genetic modifications in ergosterol synthesis pathway should result in the increase of cytosolic free NADH/NAD^+^ ratio accordingly. However, the introduction of 7-DHC biosynthesis pathway into SyBE_Sc01100001 did not show significant influence on redox imbalance, that was because the ergosterol accumulation in the strains derived from wild-type BY4742 did not change dramatically (S4 Fig). The increase of cytosolic free NADH/NAD^+^ ratio in SyBE_Sc01100010 might attribute to the production of acetate (S5 Fig), which could lead to an imbalanced redox state as well.

The increase in NADH availability favored the production of reduced metabolites [41,42], like glycerol and ethanol [4]. Nevertheless, in this study, similar concentration trend was observed between cytosolic free NADH/NAD^+^ ratio and glycerol but not ethanol. Meanwhile, a series of ergosterol supplement experiments were conducted. The results showed that glycerol did not go down as the ergosterol concentration increased (S6 Fig), indicating that glycerol was produced as a reflection of an imbalanced redox state rather than the stress response to cell damage related to ergosterol limitation. Moreover, to drive the redox chemistry to a specified direction and increase the production of our desired metabolite, it is essential to manipulate intracellular redox state as well as cofactor levels [43]. After cofactor regeneration, the cytosolic free NADH/NAD^+^ ratio (0.002±0.001) and the concentration of glycerol (0.496±0.003 g/L) of the SyBE_Sc01100020 were all below the value of wild-type BY4742 (0.003±0.001) and 0.507±0.001 g/L). However, the concentration of ethanol in SyBE_Sc01100020 was 7.788±0.035 g/L, which is still higher than the value of BY4742 (7.338±0.064 g/L). Our strategy to alleviate redox imbalance by oxidation of excess NADH showed significant influence on glycerol formation but not ethanol, which might due to that the ethanol production was redox neutral while the glycerol production was not. Thus, ethanol acted as an indirect indicator of redox state in this study.

In order to increase the productivity of our target product 7-DHC further, more strategies could be attempted. Other enzymes for cofactor regeneration such as NADH kinase (POS5) could be involved in to affect the ratio of NADH/NAD^+^ and increase the 7-DHC production. The NADH kinase was proved to be effective to alleviate redox imbalance caused by D-xylose utilization pathway in engineered *S*.*cerevisiae* strains expressing xylose reductase and xylitol dehydrogenase [44].

## Supporting Information

S1 Fig Codon optimized sequence for *dhcr24*, *acs and acl*. (TIF)

S2 Fig GC-TOF-MS analysis of strains derived from BY4742 and ΔERG5. (TIF)

S3 Fig Stoichiometric calculations for 7-DHC biosynthesis pathway. In the reactions from FPP to zymosterol, if either NADH or NADPH could be utilized, NADH or NAD^+^ were considered as the cofactor in the calculations of Eq (1).
(TIF)

S4 Fig The ratio of ergosterol accumulation to that in BY4742. The ratio of ergosterol accumulation in SyBE_Sc01100001 to that in BY4742 was 1.06(±0.03) and that of SyBE_Sc01100010 was 1.03(±0.04).
(TIF)

S5 Fig Acetate production in strains derived from BY4742 and ΔERG5. The acetate concentration produced by SyBE_Sc01100010 was 0.713(±0.005) g/L and no acetate was produced in other strains.
(TIF)

S6 Fig The change of glycerol in strains derived from ΔERG5 when different concentrations of ergosterol were supplemented into the media. (TIF)
