# Supplementary figures and images for: Alleviating Redox Imbalance Enhances 7-Dehydrocholesterol Production in Engineered Saccharomyces cerevisiae
Source: PLoS One. 2015 Jun 22;10(6):e0130840. doi: 10.1371/journal.pone.0130840 (PMC4476719; doi:10.1371/journal.pone.0130840)

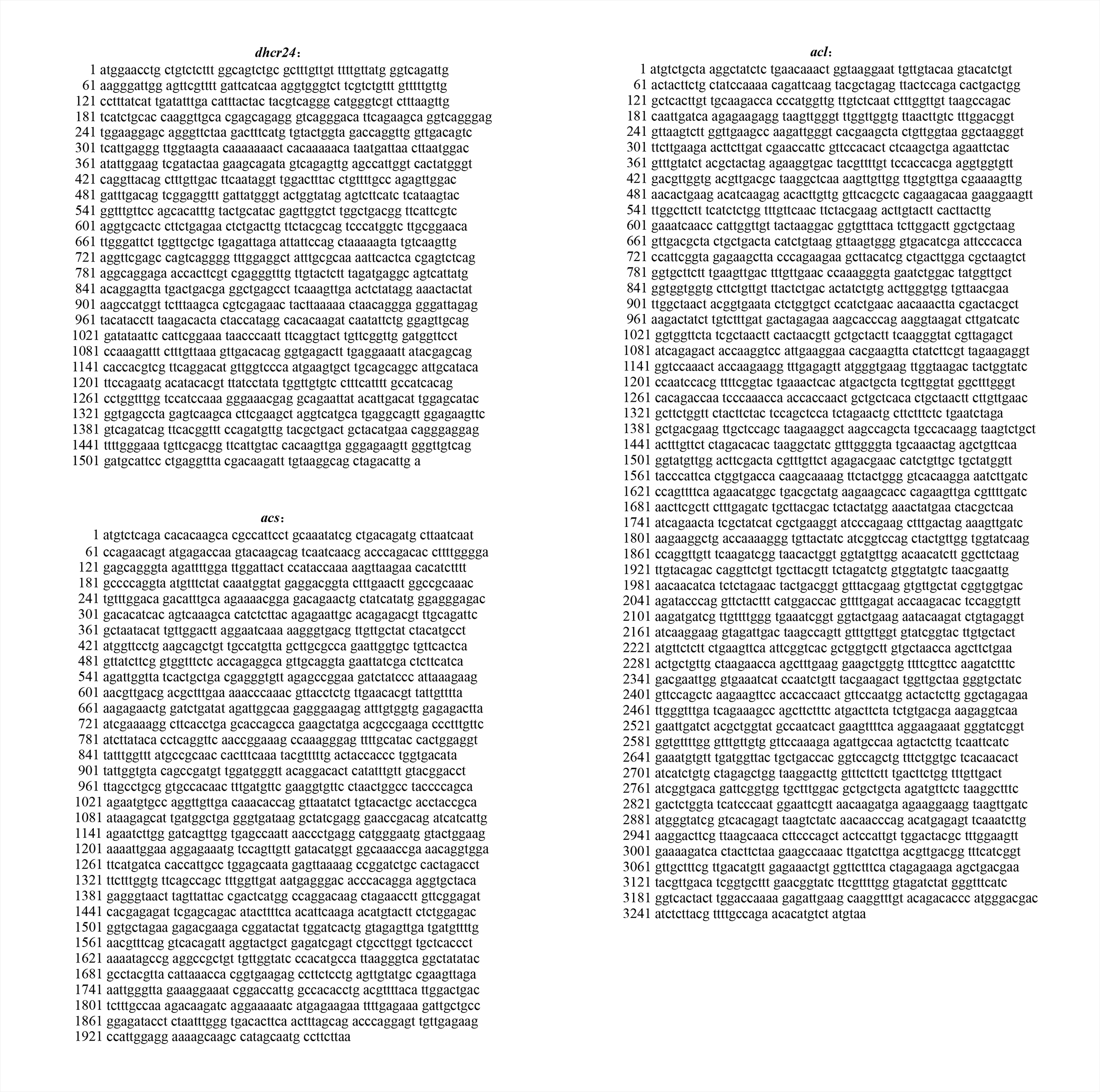

Supplement: S1 Fig — (TIF) [file pone.0130840.s001.tif]

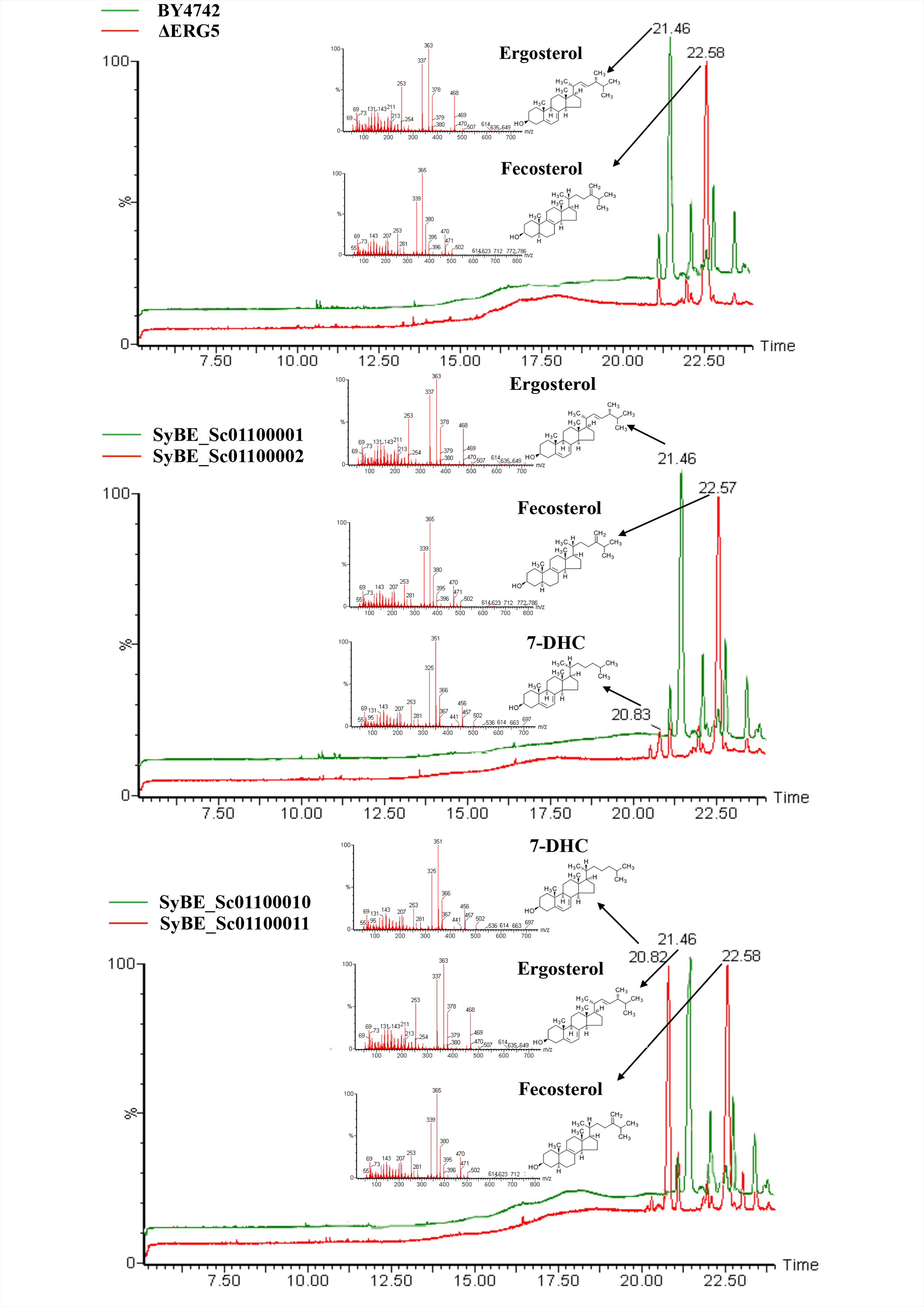

Supplement: S2 Fig — (TIF) [file pone.0130840.s002.tif]

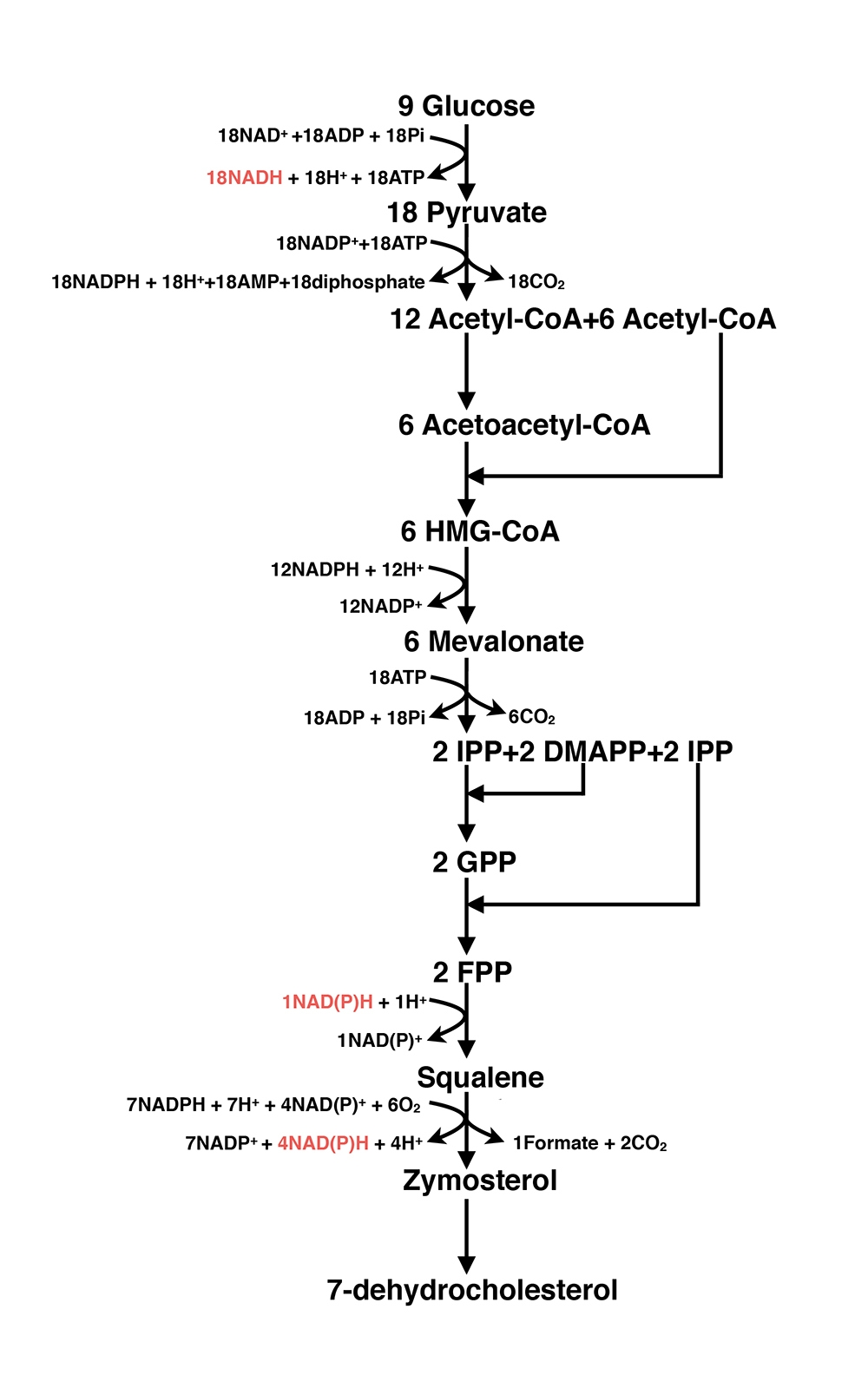

Supplement: S3 Fig — In the reactions from FPP to zymosterol, if either NADH or NADPH could be utilized, NADH or NAD+ were considered as the cofactor in the calculations of Eq (1). (TIF) [file pone.0130840.s003.tif]

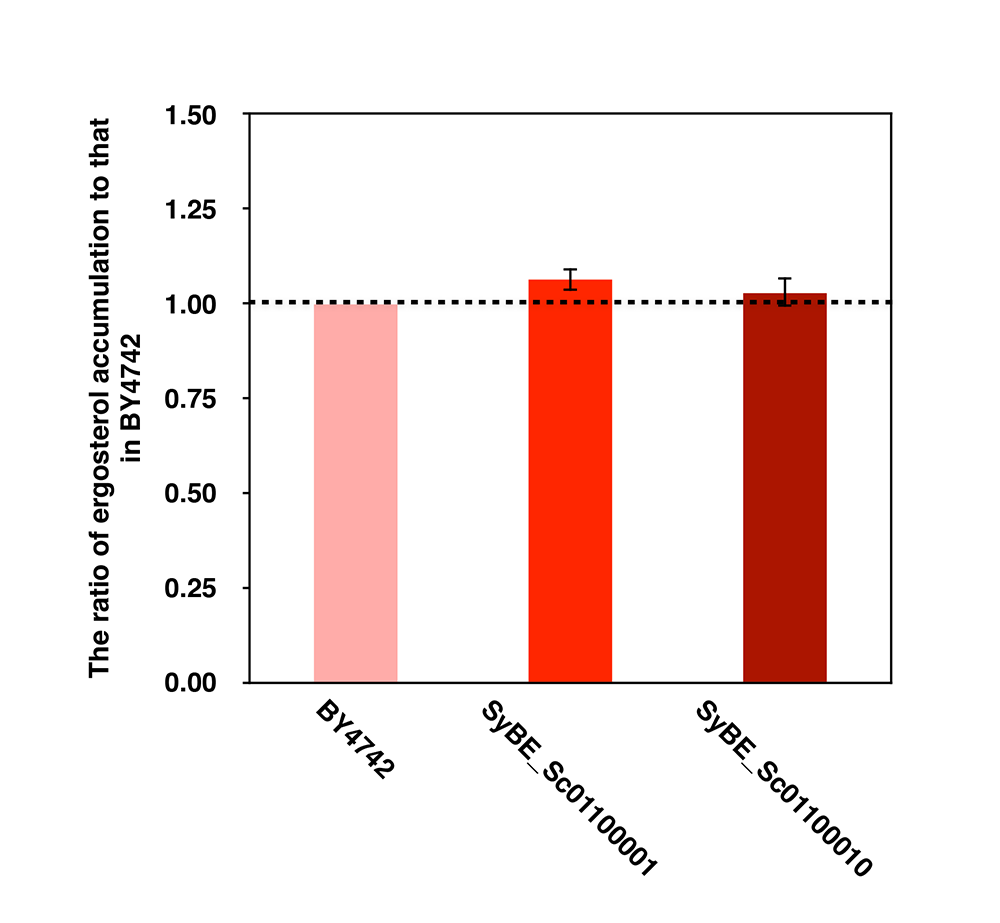

Supplement: S4 Fig — The ratio of ergosterol accumulation in SyBE_Sc01100001 to that in BY4742 was 1.06(±0.03) and that of SyBE_Sc01100010 was 1.03(±0.04). (TIF) [file pone.0130840.s004.tif]

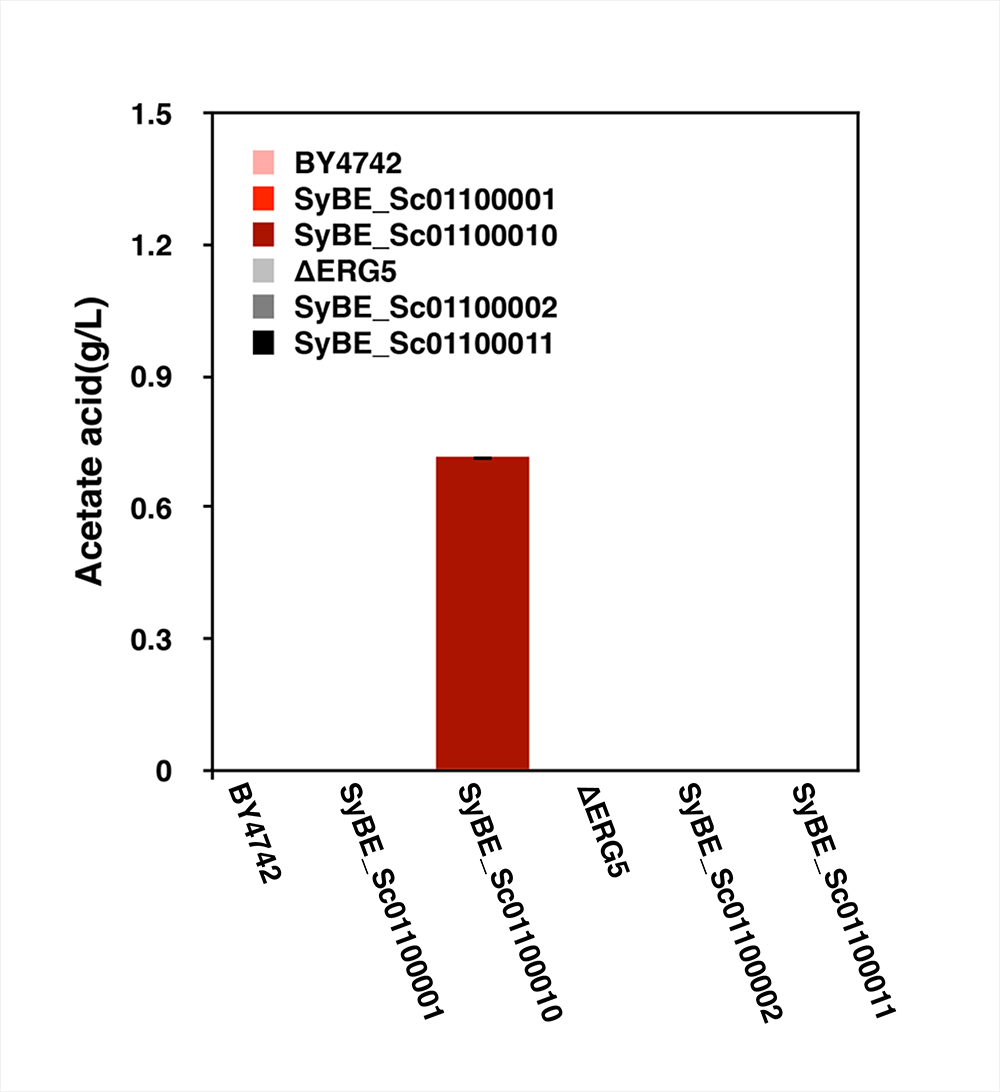

Supplement: S5 Fig — The acetate concentration produced by SyBE_Sc01100010 was 0.713(±0.005) g/L and no acetate was produced in other strains. (TIF) [file pone.0130840.s005.tif]

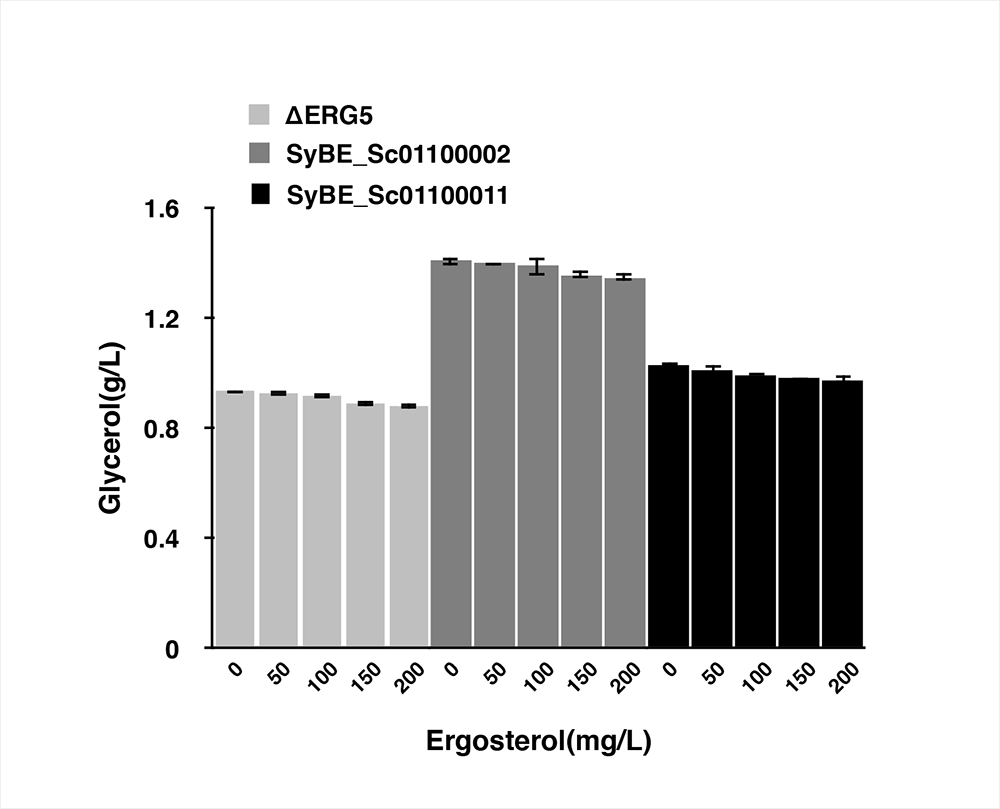

Supplement: S6 Fig — (TIF) [file pone.0130840.s006.tif]
